# Characterizing the safety and efficacy of injection pharyngoplasty as a treatment for velopharyngeal insufficiency in adults

**DOI:** 10.1007/s00405-025-09767-1

**Published:** 2025-11-12

**Authors:** Arjan Kalra, Laurence Gascon, William S. Tierney, Ian Griffiths, Rebecca C. Nelson, Paul C. Bryson

**Affiliations:** 1 Cleveland Clinic Lerner College of Medicine, EC-10 Cleveland Clinic, 9501 Euclid Ave, Cleveland, OH 44195 USA; 2 Cleveland Clinic Voice Center, Head and Neck Institute, 9500 Euclid Ave, A-71, Cleveland, OH 44195 USA

**Keywords:** Velopharyngeal insufficiency, Augmentation, Injection pharyngoplasty, Calcium hydroxyapatite, Autologous fat

## Abstract

**Purpose:**

Although injection pharyngoplasty (IP) has been well described as a treatment for velopharyngeal insufficiency (VPI) in the pediatric population, there are far fewer studies on its safety and effectiveness in adults with acquired VPI. Our study aims to characterize the safety and efficacy of IP as a treatment for acquired VPI in adults while identifying underlying VPI etiologies and injection characteristics that are associated with positive outcomes.

**Methods:**

We conducted a retrospective analysis of 30 adult patients with acquired VPI who were treated with IP. We collected data on patient demographics, etiology of acquired VPI, and IP procedural characteristics for analysis. Descriptive statistics are provided for patient demographic and IP procedural characteristics.

**Results:**

In our cohort, 16 patients had a history of radiation and/or chemotherapy while 14 patients had acquired VPI due to other etiologies. A majority of patients (*n* = 16) in our study had their VPI resolved with a single injection. Of all 61 injections recorded in our cohort, the most frequent injection agent was calcium hydroxyapatite (*n* = 35) followed by autologous fat (*n* = 15). We found that 27 of the patients in our cohort reported improvement in their acquired VPI after IP with only 9 reporting temporary minor complications that were resolved without intervention.

**Conclusion:**

Our study further establishes IP as a safe and effective procedure for adult patients with acquired VPI.

## Introduction

The velopharyngeal sphincter normally carries out the critical function of separating the nasal and oral cavities during speech and swallowing [1]. Velopharyngeal insufficiency (VPI) is a condition where the velopharyngeal sphincter is unable to effectively connect the soft palate and pharyngeal walls resulting in many functional impairments including nasal regurgitation and hypernasal speech [2]. While VPI is most commonly caused by cleft palate, this condition can occur secondary to neurological disorders, pediatric syndromes, and surgical scarring [3]. Additionally, VPI can also be an acquired clinical manifestation secondary to treatment of head and neck cancers, specifically oropharyngeal cancer and skull-based tumors [4]. Due to improvement in treatment of head and neck cancer over the past few decades, the concern for acquired VPI secondary to treatment of head and neck cancer has become increasingly relevant as more cancer patients are likely to survive longer and develop this complication during survivorship [5].

Although certain forms of VPI without structural or neurological deficits can be treated with speech therapy, the majority of VPI in adult patients is treated with surgical intervention to create a seal between the oropharynx and nasopharynx during speech [6, 7]. There are four main categories of surgical intervention for VPI: augmentation of the posterior pharyngeal wall (including injection pharyngoplasty), palatoplasty, pharyngeal flap, and sphincter pharyngoplasty [6]. While patients with larger and more severe velopharyngeal defects may require more invasive surgical procedures, these procedures may result in complications such as postoperative bleeding, flap detachment, or postoperative obstructive sleep apnea [8]. Furthermore, there is concern in radiated tissue of healing problems or access difficulties for these surgeries in patients with post-radiation trismus. Therefore, those with more moderate symptoms and defects, such as velopharyngeal gaps below 5 mm, may be better treated with less-invasive surgical procedures such as injection pharyngoplasty [8, 9].

Injection pharyngoplasty (IP) is a technique to inject materials into the posterior velopharynx to reduce the velopharyngeal gap with success having been shown with injection of autologous fat and exogenous fillers such as calcium hydroxyapatite [10]. This procedure has been well-described as a treatment for VPI in pediatric patients [11–14]. In adult VPI patients, there have been far fewer studies on the safety and efficacy of IP. The limited existing literature has described successful resolution of VPI in adult patients using autologous fat [15, 16, 18], cadaveric human skin [8], hyaluronic acid [17, 18], hyaluronic acid and dextranomer copolymer [10, 18], and calcium hydroxyapatite [18]. Given the limited research on injection pharyngoplasty for treatment of VPI in adult patients, there is a need for more studies to identify the pathologies and patient populations that may benefit from this less-invasive surgical procedure for acquired VPI. Additionally, there is also a need for more evidence of the safety of injection pharyngoplasty given the past literature identifying potential complications associated with the procedure including epidural abscess [17, 19, 20]. This study seeks to meet these gaps in the literature by describing the efficacy and safety of injection pharyngoplasty for resolution of VPI in a single-institution retrospective cohort study of a group of 30 adult patients.

## Methods

### Patient selection

This study was approved through the Cleveland Clinic Institutional Review Board under IRB #24–609. A retrospective chart review was conducted of adult patients with acquired VPI undergoing IP from 1/1/2012 to 05/01/2024 at the Cleveland Clinic Head and Neck Institute. Diagnosis of VPI was determined by the combination of symptoms of nasal regurgitation during swallowing, hyponasal speech, and/or evidence of VPI on nasopharyngolaryngoscopy. Patients were included if they were older than 18 years at time of VPI diagnosis and underwent IP at the Cleveland Clinic Head and Neck Institute. Patients were excluded if they had undergone previous treatment for VPI before they were 18 years old.

### Data collection

Patient charts were reviewed and demographic variables were collected at diagnosis of VPI. Demographic variables collected for this study included gender, age, smoking status, and radiation/chemotherapy history. Injection pharyngoplasty procedural variables were collected from operative notes for the procedure. Injection pharyngoplasty procedural variables collected for this study included number of injections, injection material, anesthesia type, amount injected, patient reported improvement in VPI symptoms, and procedural complications.

### Procedural techniques

Written informed consent was obtained; and the risks, benefits, and alternatives of the procedure were discussed in detail. Briefly, the patient is administered 4% topical lidocaine with oxymetazoline into the right or left nares. A brief, 2–3 sprays of topical Cetacaine or Benzocaine spray (Cetylite Industries, Pennsauken, NJ) is administered to the collective region comprising the base of tongue, soft palate/uvula and posterior pharyngeal wall; in some instances, some additional 4% lidocaine spray was sprayed to the same region via a finger-powered spray tip applicator. A distal chip flexible laryngoscope is placed transnasally to visualize the posterior nasopharyngeal wall and pharynx. A 22-gauge spinal needle is typically used with a small upward bend on the distal 1–2 cm. The patient is asked to gently say “ah,” and the needle is placed through the mouth and directed to posterior pharyngeal wall with scope guidance. In some instances, a tongue depressor or gauze was used to stabilize the oral tongue. Under visualization, the material is superficially/submucosally injected into the posterior pharyngeal wall at the level of Passavant’s ridge or strategically based on the pre-injection exam in the location of VPI. Care is taken to avoid deep injection into the paraspinal musculature. Intermittently, the patient is re-tested to ensure palatal closure, and additional areas are injected as needed. Post-injection, patients correspond electronically or in person at about 1 month to evaluate success of procedure and then as needed when the injection effectiveness has worn off. The procedure can be repeated as necessary.

In the OR setting, injection was also performed with transnasal endoscopic guidance. In the oral cavity, a tongue depressor was used as needed. For autologous fat, absorbable gelatin powder (Gelfoam, Pfizer, New York, New York, USA), and Alloderm (Cymetra, Lifecell Corp, Branchburg, NJ), a brunning syringe system with an 18 gauge needle was used. Fat harvest was from the lower abdomen with a small 2–3 cm incision with typical sterile technique or via liposuction around the umbilicus using a Tulip/Tenard cannula. Fat was prepared by removing fibrous attachments and washing lobules in lactated ringers solution.

### Data analysis

Descriptive statistics are provided for patient demographic and IP procedural characteristics.

## Results

### Patient demographics

Our study of 30 patients with acquired VPI treated with IP had 18 male patients and 12 female patients that were followed for an average of 22.8 months (range 1–149 months) (Table 1). The mean age of the patients in our study was 52.5 years old with the youngest patient being 20 years old and the oldest patient being 79 years old. A majority of the patients in our study sample had no smoking history (*n* = 18), with the next most common smoking status being former smoker (*n* = 7) followed by current smoker (*n* = 5). 16 patients had a history of radiation and/or chemotherapy while 14 patients had acquired VPI due to other etiologies. The non-cancer etiologies that led to acquired VPI were neurologic disorders (*n* = 5), idiopathic causes (*n* = 5), and stress VPI (*n* = 4) (Table 3). A majority of the patients in this study had their VPI resolved with only a single injection (*n* = 16). Several patients, however, required repeat procedures due to the benefits of their initial VPI fading over time; these patients required two injections (*n* = 6), three injections (*n* = 4), or four or more injections (*n* = 4) (Table 1).

**Table 1:** Patient demographic characteristics

Demographic characteristic	Number Of Patients *n* (%)
Gender	
Male	18 (60%)
Female	12 (40%)
Age	Mean = 52.5 (sd = 20.46; Range = 20–79)
Follow-Up (Months)	Mean = 22.8 (sd = 31.73; Range = 1–149)
Smoking status	
Never Smoked	18 (60%)
Former Smoker	7 (23.33%)
Current Smoker	5 (16.67%)
Radiation and/or Chemotherapy	
Yes	16 (53.33%)
No	14 (46.67%)
Number of injections	
One	16 (53.33%)
Two	6 (20%)
Three	4 (13.33%)
Four or more	4 (13.33%)

### Injection characteristics

The mean amount of material injected in our study was 2.79 cc (standard deviation 1.51) with a minimum of 0.5 cc and a maximum of 6 cc. Out of 61 total injections, the most frequent injection agent in our study was calcium hydroxyapatite (*n* = 35) (Table 2). The next most frequent injection agents in order were autologous fat (*n* = 15), hyaluronic acid (*n* = 4), silk filler (*n* = 3), absorbable gelatin powder (*n* = 2), and Alloderm (*n* = 2). There was a slightly higher amount of injections which were done under only local anesthetic (*n* = 34) than there were injections done under general anesthesia (*n* = 26) (Table 2). One injection was done under monitored anesthesia care. In this study, a vast majority of the 30 patients included in our study reported some degree of improvement of VPI after IP (*n* = 27) whereas only a few reported no improvement at all post-IP (*n* = 3) (Table 2). 9 patients in this study had pre- and post-operative modified barium swallowing study (MBSS) data available for analysis. 6 patients saw clear improvements in MBSS scoring after IP (Patients 1, 5, 8, 9, 11, 13, 18, 19, 24), 1 patient saw no improvement in MBSS scoring after IP (Patient 18), and 2 patients saw decreases in MBSS scoring after IP (Patients 11 and 24).

**Table 2:** Injection pharyngoplasty procedural characteristics

IP Procedural variable	Number of injections *n* (%)
Material for injection	
Calcium Hydroxyapatite	35 (57.38%)
Autologous Fat	15 (24.59%)
Hyaluronic Acid	4 (6.56%)
Silk Filler	3 (4.92%)
Absorbable Gelatin Powder (Gelfoam)	2 (3.28%)
Alloderm (Cymetra)	2 (3.28%)
Anesthesia type	
Local	34 (55.74%)
General	26 (42.62%)
Monitored Anesthesia Care	1 (1.64%)
Amount injected	2.79 cc (sd = 1.51; range = 0.5–6)
Patient reported improvement of VPI symptoms	
Improvement in VPI symptoms	27 (90%)
No improvement in VPI symptoms	3 (10%)
Complications	
No complications	20 (67%)
Minor complications (e.g., pain, swelling)	10 (33%)
Major complications (e.g. infection, abscess)	0 (0%)

### Complications

The majority of patients in our study reported no complications (*n* = 21) (Table 2). Those who did report complications only reported minor complications (*n* = 9). Nearly all the complications reported in this study were localized around the site of IP with the exception being abdominal fat hematoma at the donor site for autologous fat used in IP (*n* = 1, Patient 20) (Table 3). The complications reported in this study include diminished voice (*n* = 2, Patients 13 and 18), sore throat and minor, transient bleeding not requiring intervention(*n* = 1, Patient 30), and pain/soreness/discomfort (*n* = 6, Patients 2, 4, 11, 15, 24, 29). All complications reported in this study were temporary and resolved without intervention. No major complications such as infection or abscesses were observed in the patients in our study (Table 2).

**Table 3:** Study results for individual patients

					Modified barium swallow study (MBSS)				
Patient	Age	Sex	Follow up in months	VPI etiology	Pre-	Post-	Patient reported improvement	Complications	Total number of injections
1	54	F	55	Cancer Treatment	Severe oropharyngeal Dysphagia	Mild-moderate oral dysphagia. Moderate-marked pharyngeal dysphagia	Voice improved, dysarthria and hyponasality stable	None	5
2	55	M	1	Cancer Treatment	Mild-moderate oropharyngeal dysphasia; nasal regurgitation	N/A	Nasality reduced	Pain with swallowing post-IP	1
3	70	M	2	Cancer Treatment	Moderate oropharyngeal dysphagia; aspiration appreciated	N/A	Reduced nasal regurgitation	None	1
4	21	F	17	Stress VPI	N/A	N/A	Voice efficiency and tone has improved	Temporary neck soreness and difficulty swallowing post-IP	3
5	73	M	10	Cancer Treatment	Moderate oropharyngeal dysphagia; nasal regurgitation	Moderate-marked pharyngeal dysphagia; no nasal regurgitation	No further episodes of nasal regurgitation	None	1
6	77	F	3	Cancer Treatment	Severe oropharyngeal dysphasia; silent aspiration	N/A	No improvement	None	1
7	69	F	14	Cancer Treatment	Mild oral dysphagia; moderate pharyngeal dysphasia	N/A	Stronger voice; no more VPI symptoms	None	1
8	71	M	58	Cancer Treatment	Mild to moderate dysarthria; mild to moderate oral and moderate pharyngoesophageal dysphagia; risk of nasal regurgitation	Mild-moderate oral dysphagia. Moderate-marked pharyngeal dysphagia	Significant improvement in voice and resolution of nasal reflux	None	3
9	74	M	6	Cancer Treatment	Severe to profound pharyngeal dysphasia; poor velum closure with nasopharyngeal reflux	Moderate-severe oropharyngeal dysphagia	Improvement in nasality of voice and regurgitation of liquids	None	1
10	70	M	30	Cancer Treatment	Pharyngoesophageal dysphagia; silent aspiration and aspiration with reflexive cough noted	N/A	No longer has a swallowing problem	None	2
11	67	M	63	Cancer Treatment	Mild oral dysphagia; moderate pharyngeal dysphagia	Moderate oral dysphagia; severe pharyngeal dysphagia	Large improvement in VPI other than when patient sneezes	Temporary fullness the week after first IP	4
12	72	M	28	Neurologic	Oropharyngeal dysphagia with high aspiration risk	N/A	Mildly reduced nasal regurgitation; overall good benefit	None	4
13	64	M	9	Neurologic	Marked/severe pharyngeal dysphagia	Mild oropharyngeal dysphagia; functional swallow with good airway protection	Swallowing improved with less phlegm and near absence of nasal regurgitation	Temporary diminished voice post-IP	1
14	72	M	3	Cancer Treatment	Mild oral dysphagia; moderate-marked pharyngeal dysphagia	N/A	Voice is stable and reduced nasal regurgitation	None	1
15	24	M	16	Neurologic	N/A	N/A	Does not feel symptomatic VPI, voice is doing well with noticeable change perceived by others	Temporary pain and discomfort while talking post-IP	3
16	50	F	18	Cancer Treatment	Moderate nasopharyngeal dysphagia with nasopharyngeal regurgitation	N/A	Had reduced symptoms immediately after injection	None	3
17	79	F	3	Cancer Treatment	Oropharyngeal dysphagia; high risk of aspiration	N/A	Reduced nasal regurgitation and VPI symptoms	None	2
18	55	F	28	Neurologic	Mild-moderate pharyngeal dysphagia to liquids; moderate-severe dysphagia to solids	Moderate/marked pharyngeal deficits persist	Nasal regurgitation was improved	Temporary diminished voice post-IP	1
19	47	M	38	Neurologic	Severe pharyngeal dysphagia; trace VPI with liquids	Moderate-severe pharyngeal dysphagia persists; occasional episodes of trace aspiration with thin liquid	VPI symptoms improved	None	1
20	30	F	4	Idiopathic	N/A	N/A	Voice is slowly improving; no VPI symptoms	Abdominal fat hematoma at donor site	1
21	28	M	81	Cancer Treatment	Mild/moderate pharyngeal dysphagia with mild nasopharyngeal regurgitation	N/A	Voice remains strong with no symptoms of nasopharyngeal regurgitation and improved VPI	None	1
22	73	M	2	Idiopathic	N/A	N/A	Voice slowly recovering; no longer has nasal emission of air	None	1
23	20	M	3	Stress VPI	N/A	N/A	No improvement	None	2
24	48	F	10	Idiopathic	Mild oropharyngeal dysphagia	Moderate-severe oral dysphagia; mild-moderate pharyngeal dysphagia	Voice and VPI immediately improved	Pain and could not turn neck the first week post-IP	2
25	27	F	7	Idiopathic	Moderate pharyngeal dysphagia	N/A	No improvement	None	1
26	22	F	14	Stress VPI post-surgery	N/A	N/A	No issues with air leaking and patient is very happy with results of injection	None	2
27	48	M	149	Cancer Treatment	Moderate-severe pharyngeal dysphagia with risk for aspiration with larger bolus sizes	N/A	Patient had temporary benefit from injections with improvement of VPI symptoms	None	8
28	63	M	3	Cancer Treatment	Oral and pharyngeal phase dysphagia	N/A	Had 8 weeks of benefit in air escape and swallowing but noticed some fading of improvements	None	1
29	31	M	8	Idiopathic	N/A	N/A	Improvement in nasality of his speech	Temporary significant discomfort while lying flat	2
30	21	F	1	Stress VPI	N/A	N/A	No significant postoperative pain and patient is doing much better	Sore throat and slight bleeding day after IP	1

## Discussion

VPI can be a debilitating condition that greatly affects patients’ quality-of-life; in this study we have demonstrated that it can be effectively and safely treated using IP with a variety of materials. Although injection pharyngoplasty has been described in the literature as early as 1968 [21], the vast majority of literature supporting its use as a treatment for VPI has emerged in the past two decades due to more diagnostic awareness of VPI and greater interest and adoption in awake/non-sedated procedures as well as the emphasis on quality-of-life in head-and-neck cancer survivorship. This literature has been limited to smaller case series [8, 10–16], with one notable exception documenting effective use of posterior pharyngeal augmentation in 111 patients from 1968 to 2008 [22]. Successful improvements in VPI in these studies has ranged from 57 to 100% of patients [8, 10–16, 22]. Our study fits within this range with improvement in VPI in 90% of our patients. An important caveat is that this literature is highly variable as to their preferred outcome measure with studies using either patient reported improvement [11, 12, 14, 16], speech nasality/performance [10, 13–15, 22], velopharyngeal gap size [10, 15], or dysphagia/swallowing diagnostic tools including the MBSS score or eating assessment tool (EAT-10) [8]. Although we found improvements in MBSS scores in 67% of patients who had pre- and post-operative MBSS scores available, our study utilized patient reported improvement as a primary outcome measure given the lack of consistent availability of MBSS scores for the majority of our patients. We also recognize that VPI is often one of several neurological or tissue deficits in this patient population. Given that improvement in VPI may not change overall pharyngeal dysphagia and that other standardized scoring systems such as EAT-10 were not specifically designed for VPI, we relied more on subjective patient reported improvement post-injection.

In alignment with the existing literature, our study demonstrated that VPI of several different etiologies can be successfully treated using IP with a variety of materials including calcium hydroxyapatite, autologous fat, hyaluronic acid, silk filler, absorbable gelatin powder (Gelfoam), and Alloderm (Cymetra). Importantly, the 3 patients in our study who had saw no improvement all had received different materials for their IP implying that their lack of improvement was more likely a result of tissue characteristics and global swallow function rather than a result of the type of material they received in their IP. Additionally, while past studies have identified a variety of major complications (i.e., abscess [17, 19]) and minor complications (i.e., neck pain [12, 13]) associated with IP, we only saw minor complications in our study. This adds further evidence to the safety of IP as a treatment for VPI. Finally, we have showed a high rate of successful IP procedures under both general anesthesia and local anesthetic further supporting the idea that this procedure can be done both in an in-office or operating room setting [8].

This study adds to the existing literature demonstrating that VPI of diverse etiologies in adult patients can be safely and effectively managed with IP using a variety of materials under both general and local anesthesia. Specifically, this study stands out as the largest sample of adult patients to demonstrate the safety and efficacy of IP as a treatment method for VPI. We suspect more outcomes will be published as the importance of head and neck cancer survivorship is highlighted in the literature.

This study also has certain limitations to acknowledge. First, due to the retrospective nature of this study we were unable to ensure that all patients had pre- and post-operative MBSS data collected prior to their IP. Having data from this standardized diagnostic tool for dysphagia could improve characterization of the degree of improvement provided by IP for VPI. Second, although this study was aimed at investigating factors that determine successful resolution of VPI using IP, it is possible that selection of patients with more milder forms of VPI (previously defined as velopharyngeal gaps less than 5 mm [22]) for IP rather than other surgical interventions may have biased the study results to have a higher effectiveness due to patients that are optimal for the procedure undergoing IP. Likewise, patients with more severe pharyngeal dysphagia and poor oral intake may have more decreased quality of life at baseline and closure or VPI may not have added substantial value to their swallow related quality of life.

In 2020 Denadai called for further research with larger samples to best understand which patients are best suited for treatment of their VPI with IP using autologous fat as well as the optimal IP procedural technique [23]. While this study answers that call by demonstrating that IP can resolve VPI effectively using a wide variety of materials including autologous fat, it also highlights the need for further studies that investigate whether IP can resolve VPI effectively using standardized outcome measures such as MBSS and studies that investigate best circumstances for each type of material for IP.

## Conclusion

In our retrospective cohort study of 30 adult patients with acquired VPI of diverse etiologies treated using IP with a wide variety of injection materials, we found 90% improvement in VPI symptoms and only 30% of patients reporting minor complications. This study further establishes IP as a safe and effective procedure for adult patients with acquired VPI.
